# Transcranial Electromagnetic Treatment Stops Alzheimer’s Disease Cognitive Decline over a 2½-Year Period: A Pilot Study

**DOI:** 10.3390/medicines9080042

**Published:** 2022-08-03

**Authors:** Gary Arendash, Haitham Abulaban, Susan Steen, Ross Andel, Yanhong Wang, Yun Bai, Rob Baranowski, Jon McGarity, Lyle Scritsmier, Xiaoyang Lin, Ning Shen, Ali Aljassabi, Yitong Li, Chuanhai Cao

**Affiliations:** 1 NeuroEM Therapeutics, Inc., 13231 N. 14th Place, Phoenix, AZ 85022, USA; jonmcgarity@gmail.com (J.M.); scrits@cox.net (L.S.); 2 Axiom Clinical Research of Florida, 2919 W. Swann Ave, Tampa, FL 33609, USA; habulaban.axiom@gmail.com (H.A.); ssteen.axiom@gmail.com (S.S.); 3 Byrd Alzheimer’s Institute, University of South Florida, Tampa, FL 33613, USA; 4 School of Aging Studies, University of South Florida, Tampa, FL 33620, USA; randel@usf.edu; 5 MegaNano Biotech, 3802 Spectrum Blvd., Tampa, FL 33612, USA; yanhongwang@usf.edu (Y.W.); ybai@megananobiotech.com (Y.B.); xlin3@usf.edu (X.L.); alialjassabi@usf.edu (A.A.); yitongli@usf.edu (Y.L.); 6 Taneja College of Pharmacy, University of South Florida, Tampa, FL 33612, USA; 7 Left Coast Engineering, Escondido, CA 92025, USA; rob@leftcoasteng.com; 8 School of Arts & Science, University of South Florida, Tampa, FL 33621, USA; ningshen@usf.edu

**Keywords:** Alzheimer’s disease, long-term transcranial electromagnetic treatment, cognitive benefits, AD markers

## Abstract

**Background:** There is currently no therapeutic that can stop or reverse the progressive memory impairment of Alzheimer’s disease (AD). However, we recently published that 2 months of daily, in-home transcranial electromagnetic treatment (TEMT) reversed the cognitive impairment in eight mild/moderate AD subjects. These cognitive enhancements were accompanied by predicted changes in AD markers within both the blood and cerebrospinal fluid (CSF). **Methods:** In view of these encouraging findings, the initial clinical study was extended twice to encompass a period of 2½ years. The present study reports on the resulting long-term safety, cognitive assessments, and AD marker evaluations from the five subjects who received long-term treatment. **Results:** TEMT administration was completely safe over the 2½-year period, with no deleterious side effects. In six cognitive/functional tasks (including the ADAS-cog13, Rey AVLT, MMSE, and ADL), no decline in any measure occurred over this 2½-year period. Long-term TEMT induced reductions in the CSF levels of C-reactive protein, p-tau217, Aβ1-40, and Aβ1-42 while modulating CSF oligomeric Aβ levels. In the plasma, long-term TEMT modulated/rebalanced levels of both p-tau217 and total tau. **Conclusions:** Although only a limited number of AD patients were involved in this study, the results suggest that TEMT can stop the cognitive decline of AD over a period of at least 2½ years and can do so with no safety issues.

## 1. Introduction

Despite billions of dollars having been spent and over 20 years of failed clinical trials, there remains no therapeutic intervention capable of stopping or reversing the debilitating cognitive decline of Alzheimer’s disease (AD) that occurs over a 2–20-year period. Approximately 50 million people are living with AD worldwide, with that number projected to exceed 152 million by 2050 if an effective treatment is not found to stop or reverse the disease [1]. Someone in the world develops AD or dementia every three seconds [1], and the disease, if unabated, will cost the U.S. alone over USD 1 trillion by 2050 [2].

Since the early 2000′s, over 150 “pharmaceutical” therapies have been clinically evaluated, with all of them failing to stop or reverse the cognitive decline of AD. Many AD researchers have now argued that this lack of drug efficacy is due to beginning treatment too late in the AD process and that focus should now be placed on prevention-based AD clinical studies [3]. However, we believe it is more likely that most current AD drugs are flawed in that (1) they have minimal or no bioavailability within the brain’s neurons [4], and (2) they do not target the primary culprits of AD, which recent studies indicate are small toxic “oligomers” of β-amyloid (Aβ) and tau [5,6,7,8,9,10,11,12]. These small soluble aggregates are formed inside neurons, where they cause neuronal dysfunction and death [13,14,15,16,17,18,19,20,21,22]. Thus, any AD therapeutic hoping to stop or reverse AD cognitive decline will probably need to penetrate the blood–brain barrier, then the brain’s neurons, and be able to disaggregate BOTH Aβ and tau oligomers, especially after the disease has become established.

We have developed a new bioengineering-based, proprietary technology against AD called transcranial electromagnetic treatment (TEMT). This non-pharmacologic therapeutic involves administering electromagnetic waves (in the radiofrequency range) that easily penetrate the bony cranium and effortlessly enter all neurons throughout the entire forebrain. In preclinical studies involving AD transgenic mice, we have shown the ability of long-term TEMT to prevent or reverse cognitive impairment [23,24,25]. The primary mechanisms for these cognitive benefits appear to be the disaggregation of toxic Aβ oligomers (both inside and outside neurons) and an associated mitochondrial enhancement inside neurons [13,23]. We have most recently subjected human AD brain tissues to TEMT and found disaggregation of not only Aβ oligomers but also tau oligomers [26]. Thus, TEMT can effectively deal with both culprits of AD.

To translate these studies clinically, we designed, developed, and fabricated a first-of-its-kind proprietary head device called the MemorEM^TM^ for in-home use to administer TEMT to AD subjects by their caregivers. In an initial pilot clinical trial involving eight mild/moderate AD subjects, twice daily 1 h TEMT was provided in-home for 2 months [27]. Following this treatment period, a reversal of cognitive impairment in key tasks was observed for seven of the eight subjects with the ADAS-cog and for all eight subjects with the Rey AVLT [27]. Moreover, 2 months of daily TEMT provided enhanced functional MRI imaging (i.e., greater “functional” neuronal connectivity) in all AD subjects and induced changes in the Aβ isoforms in the CSF and plasma that are consistent with Aβ disaggregation in the brain [27]. The combination of extensive preclinical studies and this original clinical study’s findings resulted in the FDA providing “Breakthrough” designation for TEMT technology and the MemorEM device against AD in 2020, with the MemorEM being the first drug or device receiving that designation.

Because of the promising results from the aforementioned pilot clinical study, and the enthusiasm of the study’s AD patients/caregivers to continue treatment, 4-month and 12-month extension studies were performed, with breaks of 8 and 5 months interposed with no treatment (31 months total). Thus, the primary objective of this study was to determine the cognitive effects and safety of TEMT given over an extended period. Despite the two lengthy periods of no treatment and the limited number of subjects, the present study describes the apparent ability of daily in-home TEMT to safely stop an otherwise certain cognitive decline over a 2½-year period in six cognitive/functional tasks as well as no change in the GDS stage of AD by a caregiver assessment. In addition, long-term TEMT either decreased, increased, or modulated/rebalanced multiple AD markers in both the CSF and plasma.

Our results strongly argue for the initiation of a placebo-controlled, larger clinical trial with TEMT to treat established AD. As such, a phase IIb/III (pivotal) clinical trial will be initiated later this year.

## 2. Materials and Methods

### 2.1. Subjects

Eight subjects with mild-moderate AD were enrolled in the initial 2-month clinical protocol of this multiprotocol study over the period of early 2018 through mid-2018 at the University of South Florida Health/Byrd Alzheimer’s Institute (Tampa, FL). Subjects were required to be diagnosed with mild or moderate AD according to the National Institute of Neurological and Communicative Disorders and Stroke-Alzheimer’s Disease and Related Disorders Association (NINCDS-ADRDA) criteria. Subjects were at least 63 years of age and had an MMSE (Mini-Mental State Exam) score of 16–26 at screening. In addition, subjects had to have a Hachinski test score lower than 4 and a Global Deterioration Score above 2. If subjects were being medicated with a cholinesterase inhibitor and/or memantine, they needed to have been taking the medication(s) for at least 3 months prior to screening and had to be maintained on their present dose throughout the period of this study. All subjects gave their consent to be in this multiprotocol study, in strict accordance with the informed consent regulations of the USF Health/Byrd Alzheimer’s Institute and Axiom Clinical Research of Florida (Tampa).

Of the original eight AD subjects that completed the initial 2-month clinical protocol, five continued into the extension protocol treatment, with the remaining three subjects either not being locally located (one subject), not continuing because of unrelated health reasons (one subject), or having too long of a period between the original protocol and the extension study I treatment (one subject). For each of these five subjects participating in this multiprotocol study, the demographics and characteristics are shown in Table 1. In addition to the diagnosis of AD from the aforementioned cognitive assessments at screening/baseline, AD diagnosis was further validated by three indices included in this table, with detailed descriptions in the published results from our initial pilot study [27]. First, anatomic MRIs at screening indicated the presence of hippocampal/temporal lobe atrophy, and/or global cortical atrophy. Second, a quantitative analysis of FDG-PET scans at baseline indicated glucose hypometabolism in three brain areas that were averaged to provide an “AD signature meta-ROI ratio” [28], with an abnormal AD ratio being defined as ≤1.32 (90% AD sensitivity) [29,30]. Third, the CSF Aβ1-42/t-tau ratio at baseline was calculated for each subject. The mean ratio of 1.01 was below the mean ratio of 1.30 reported for diagnosed AD subjects and far below the mean ratio of 3.96 reported for aged controls [31].

The full lists of both the inclusion and exclusion criteria for this multiple-protocol study are indicated in our initial published paper on the initial 2-month protocol [27] and in all three of the aforementioned clinical protocols listed on http://www.clinicaltrials.gov (accessed on 10 May 2022) (NCT02958930, NCT03927040, and NCT04271163). For each AD subject and clinical protocol, a caregiver (spouse, family member, etc.) with non-impaired mental abilities/motor skills needed to be identified to be responsible for administering the daily treatments to the subject, keeping a diary of health measures they collect on the subject at home, and logging the patient’s condition.

### 2.2. Investigational Device

**Figure 1:**
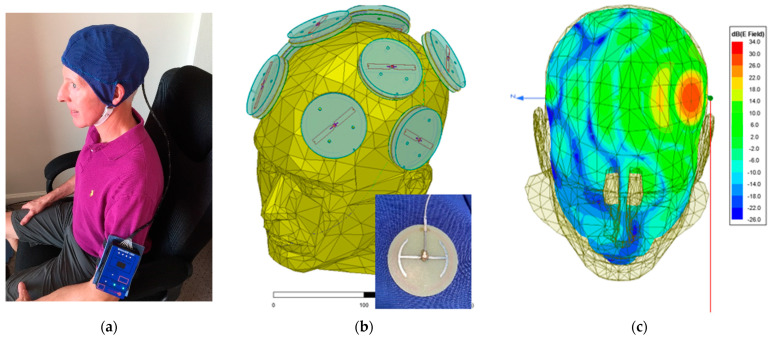
(**a**) A MemorEM^TM^ device being worn by a 63-year-old individual. The control panel/battery box is worn on the upper arm and is wired via a cable to eight electromagnetic emitters in the head cap. (**b**) The position of the eight electromagnetic emitters (four on each size of the head) embedded between the device’s two-layered head cap. A single emitter attached to its electromagnetic conduction wire is shown in the insert. Emitters collectively provide full forebrain TEMT via rapid sequential activation. (**c**) An FDTD (ANSYS) brain simulation of electric field strength showing the penetration and distribution from a single “ON” emitter at an SAR power level of 1.6 W/kg.

The proprietary MemorEM device (NeuroEM Therapeutics, Phoenix, AZ, USA) allows for complete mobility in performing most daily activities during in-home daily treatment. The device has a rechargeable battery inside the box housing and a custom-engineered circuit board in addition to a control panel on the outside of the housing for controlling treatment. Contained within the box housing is a custom-engineered circuit board/battery, which has a control panel on its surface. The box is typically worn on the upper arm and is permanently wired via a cable to eight Egyptian axe emitters (four on each side of the brain) within a two-layered head cap (Figure 1a,b). The emitters are activated sequentially at 217 Hz so that only one emitter is active at any given time. When active, an emitter projects electromagnetic waves into the brain at 915 MHz and a 1.6 W/kg power level. At this frequency and power level, FDTD (ANSYS) human head computer simulations show that the eight emitters collectively provide penetrating TEMT to the entire human forebrain, including the cerebral cortex, underlying subcortical structures (e.g., the hippocampus), and both superficial and deep cerebral vessels, albeit at decreasing power with increased depth (Figure 1c). The MemorEM device is designed to allow no more than two 1 h treatments within any given 24 h period and requires that at least a 7 h interval occurs between those two daily treatments. In December 2016, the MemorEM device was approved as a “non-significant risk” (NSR) by the Western Institutional Review Board (WIRB). In March 2020, the MemorEM device was then designated by the FDA as its first “Breakthrough Therapeutic” for the treatment of AD.

### 2.3. General Protocol

**Figure 2:**
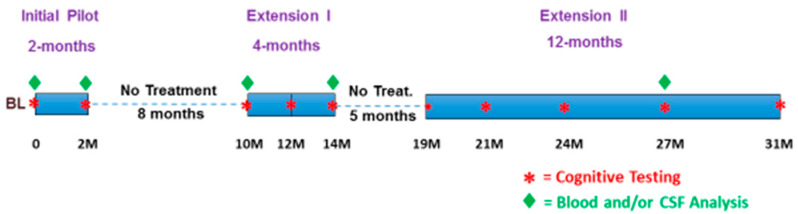
The treatment (blue bars), cognitive testing, and blood/CSF analyses for this 31-month study of TEMT safety and efficacy.

This clinical study comprised three separate but nearly identical clinical protocols (Figure 2), all involving AD patients in the initial 2-month clinical study and all approved in the U.S. by the Western Institutional Review Board (now the WCG-IRB). The initial 2-month pilot clinical protocol (ClinicalTrials.gov NCT0295830) was followed by a 4-month extension I protocol (ClinicalTrials.gov NCT03927040) and by a 12-month extension study II (ClinicalTrials.gov NCT04271163). Thus, the three clinical protocols were combined in series as a single long-term study with a 31-month duration, including two periods of no treatment. The cognitive assessments at any given timepoint in any of these three protocols were identical, as were all the blood and CSF sample collection processes in all three protocols; all blood and CSF samples were analyzed collectively at the end of the 31-month study for AD markers (see below). Following the screening and baseline clinical visits, succeeding visits occurred at 2, 10, 14, 19, 21, 24, 27, and 31 months into treatment.

### 2.4. Subject Screening

Potential subjects were given both a neurologic exam and a physical exam as well as an MMSE, the Hachinski Test, and a Global Deterioration Scale assessment. In addition, a 3-Tesla “anatomic” MRI brain scan (susceptibility-weighted imaging, or SWI, and axial FLAIR sequences) was taken for the determination of any pre-existing brain abnormalities (e.g., tumors, cerebrovascular disease/infarction, demyelinating diseases, an excessive number of brain microhemorrhages, etc.) and to serve as the baseline for any treatment effects on MRI-related endpoints. All of the above screening events required several clinical visits.

### 2.5. Baseline Evaluations

The baseline consisted of three days of evaluation/testing, all within one week of TEMT initiation in the initial 2-month pilot protocol and all scheduled during the morning hours. One day involved an FDG-PET scan being performed, while another day included functional MRI scanning (DTI) for functional connectivity. The third day involved a clinical office visit during which a comprehensive battery of cognitive tasks was administered to establish baseline cognitive performance. These tasks involved the principal measure of efficacy, the ADAS-Cog13 (maximum poor score of 85 points), and secondary measures including the Rey Auditory Verbal Learning Test (Rey AVLT), Alzheimer’s Disease Cooperative Study–Activities of Daily Living (ADL), and Digit Span Forward/Backward. These are all well-established protocols that are easily found on the internet. In addition, a baseline suicide ideation score (via the Columbia Suicide Severity Rating Scale) and a baseline Adverse Event Assessment were obtained. On one of the above three days, a 20 mL blood sample and a 15 mL CSF sample (via spinal tap) were taken for later AD marker analyses and *APOE* genotyping. Parenthetically, the effects of TEMT on FDG-PET and fMRI imaging were reported in an earlier publication involving only the initial 2-month protocol [26]. No further FDG-PET or fMRI imaging was performed in the latter two extension protocols.

### 2.6. The 31 Months of Treatment/No-Treatment Periods

The first TEMT for the initial 2-month treatment protocol was performed in the morning at the clinic, during which the subjects’ caregivers were instructed on the proper procedure for administering TEMT to the subject at home. An “Instructions for Use” manual was provided to each caregiver, who was also instructed on how and when to take blood pressure measurement with a supplied fully automated BP device as well as how and when to take body temperature with a supplied forehead-based thermometer. Also provided to caregivers was a “Patient Daily Diary” for them to enter each day’s blood pressure and temperature readings, check off daily activities (e.g., eating/drinking) as normal or different, and for them to comment on any different behaviors or undesirable side effects that may have occurred to the patient during or after that day’s treatments. A second late-afternoon TEMT administration was administered at the patient’s home by the caregiver. Throughout the following 2-month period, subjects were given twice-daily in-home TEMT treatment for 1 h each (early morning and late afternoon), as administered and overseen by the caregiver. Subjects returned to the clinic at 2-months into TEMT treatment for the same cognitive, AD biomarker, and brain imaging endpoints taken at baseline. Following an approximately 8-month period of no treatment, an extension I protocol of 4 months was initiated with once-daily 1 h treatments. Following an ensuing 5-month period of no treatment, an extension II protocol of 12 months was initiated, with twice-daily 1 h treatments for the first two months, followed by once-daily 1 h treatments for the remaining 10 months. Blood samples were analyzed from the baseline, 14 M, and 27 M samples. CSF samples were taken via spinal tap at baseline, 2 M, 10 M, and 14 M (Figure 1).

### 2.7. Patient Monitoring and Safety

The primary safety measure was an Adverse Event Assessment implemented during every clinical visit beginning with the baseline. Secondary safety measures of vitals were also collected at the same clinical visit time points (e.g., temperature and blood pressure). The secondary safety measure of suicidal tendencies was evaluated at baseline and at intervals during treatment. Throughout the TEMT administration periods, subjects were monitored by their caregivers for any undesirable side effects of treatment, including any different behaviors during/after treatment. An associated safety measure involved the diaries kept by caregivers, which included frequent blood pressure/temperature measures from the subject, a checklist of whether activities were normal/abnormal, and the notation of any unusual behavior exhibited by the subject. A final safety measure for determining any adverse effects of TEMT involved anatomic MRI scans taken at 2 months, 19 months, and 31 months into treatment for the determination of any unusual occurrences in the brain during treatment (e.g., tumors, cerebral microhemorrhages, or general volumetric changes in brain structures/lateral ventricles).

### 2.8. Blood and CSF Processing/Analysis

All 20 mL blood samples (collected at baseline and during treatment) were divided into two 10 mL BD k2-EDTA tubes and centrifuged at 300× *g* for 10 min. The upper plasma layer from each tube was transferred into a new 15 mL tube, then centrifuged again at 2000× *g* for 10 min. Then, 1 ml volumes of the top plasma layer were aliquoted into 1.5 mL tubes and stored at −80 degrees C for analysis at some future time. The two 15 mL samples of CSF collected at baseline and on day 60 were each aliquoted into 1.5 mL tubes, then frozen and stored at −80 °C until analysis of the same AD markers as indicated for plasma. At the end of the 31-month study, the plasma/CSF samples were thawed completely on ice, then mixed well on a vortex and centrifuged at 2000× *g* for 10 min to precipitate any debris for the determination of the following AD biomarkers in duplicate: soluble/monomeric Aβ1-40 and Aβ1-42, oligomeric Aβ, total tau (t-tau), and p-tau.

### 2.9. Human C-Reactive Protein (CRP) Determination

The concentration of C-reactive protein (CRP) was measured by the C-reaction-specific sandwich ELISA kit (Invitrogen Cat: KHA0031). In brief, plasma samples were diluted to 1:3000 with standard diluent buffer, CSF samples were diluted to 1:100 with standard diluent buffer, and standards were diluted at series dilution with standard diluent buffer according to the protocol; then, 100 μL of diluted standard and samples were added into the appropriate wells. The plate was then incubated for 2 h at 37 °C. After washing, 100 μL of biotin-conjugated anti-human CRP solution was added to each well and incubated for 1 h at RT. After washing, 100 μL of streptavidin HRP solution was added to each well and incubated for 30 min at RT. The plate was washed four times, and TMB peroxidase substrate was added to each well and incubated at RT for 30 min. The reaction was stopped by adding 100 μL of stop solution. The absorbance at 450 nm was read with a BioTek Synergy H4 microplate reader. The concentration was calculated based on the standards.

### 2.10. Human Aβ1-40, Aβ1-42, and Oligomeric Aβ Determination (9A9)

Antibodies (goat anti-human Aβ1-42- and goat anti-human Aβ1-40-specific antibodies) were purchased from MegaNano Diagnostics Inc. Tampa FL, while a 9A9 antibody that has specific binding to aggregated Aβ (MegaNano Diagnostics Inc., Tampa, FL, USA) and has been validated with the oligomer-specific antibody (A8) was prepared by our collaborator [32]. Instructions were followed according to those provided. The standard, detection antibody, anti-rabbit IgG HRP, and wash buffer were all prepared according to the instructions. For each well, 50 μL of standard and plasma or CSF (1:100 diluted for Aβ determinations) samples were added to the appropriate wells, then 50 μL of detection antibody for Aβ 1-40, Aβ1-42, or 9A9 (Aβ oligomers) was added to each well. Incubation occurred overnight at 4 °C with shaking. After four washes, 100 μL of diluted anti-rabbit IgG HRP was added to each well, followed by incubation for 1 h at room temperature with shaking. This was followed by four washes with wash buffer and the addition of 100 μL of stabilized chromogen to each well to allow the reaction for 10 min. Stop solution (100 μL/well) was then added, and plates were read on a BioTeck Synergy H4 reader.

### 2.11. Human Total Tau (t-tau) and Phospho-Tau (p-tau) Determinations

For human total tau, the instructions were followed according to those provided for the Thermo Fisher Human Tau (total) kit (Cat: KHB0041). The standard, streptavidin-HRP, and wash buffer solutions were prepared according to the instructions. For each well, 100 μL of standard and plasma or CSF sample (undiluted) were added, incubated overnight at 4 °C with shaking, then washed four times with wash buffer. A detection antibody (100 μL/well) was then added, followed by incubation for 1 h at room temperature. Plates were washed four times with wash buffer, then 100 μL of diluted streptavidin-PE was added to each well, followed by incubation for 1 h at room temperature with shaking. Next, plates were washed four times, followed by the addition of 100 μL of stabilized chromogen to each well. The reaction was allowed to occur for 10 min, then 100 μL of stop solution was added to each well, followed by plate reading on a BioTek Synergy H4 reader.

For human p-tau217, we used a dot blot assay to detect the plasma and CSF levels with an anti-Human p-tau217 antibody. Moreover, recombinant Tau was used to generate a standard for quantification. For each dot, 2 μL of standard at 1 μg/mL and 1 μL of plasma diluted into 200 μL or 20 μL of CSF sample at 1:10 dilution were added into each well with vacuum. An NC membrane was then blocked with blocking buffer at 4 °C for 24 h, then washed four times with wash buffer. The membrane was incubated with anti-p-tau217 antibody (1:1000) for 45 min at RT. An anti-human HPR antibody was then added after three washes and incubated for 1 h at room temperature. Blots were washed four times, then diluted with anti-rabbit IgG HRP (1:1000), followed by 1 h of incubation at room temperature with shaking. Blots were then washed four times, followed by the addition of an ECL reaction to occur for 5 min. The membranes were then detected with an imager. The dots on the film were quantified using image J software, and the amount of each sample was determined against the standard.

### 2.12. Statistical Analyses

**Figure 3:**
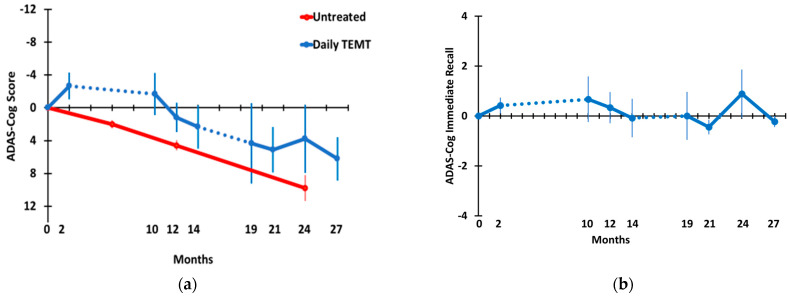
(**a**) ADAS-cog overall and (**b**) ADAS-cog immediate memory through 27 months. Blue data points and lines show performance of AD subjects given TEMT. Dotted lines indicate the 8 M and 5 M periods of no treatment. For ADAS-cog overall, the red data points and SEMs are from another clinical study evaluating ADAS-cog longitudinally in untreated AD subjects.

The change in cognitive/functional scores was analyzed using exploratory mixed-effects models. These models estimate both fixed and random effects, allowing for a superior fit of the model to the data compared to a repeated measures analysis of variance [33]. These models also allow for the inclusion of participants with missing data and are flexible in terms of covariance structure. For comparisons of baseline vs. 27 or 31 M cognitive performance and for all AD marker analyses in the plasma and CSF, paired t-tests were utilized. In the cognitive measure analyses, one subject was the sole non-performer on the ADAS-cog during the initial 2-month pilot study ([27]; Figure 3A) and was a later non-performer on the MMSE and ADL. That subject’s data were therefore not included in the analysis of these three tasks. A second subject dropped out of the study during the second 5-month non-treatment period. Although nearly all time points for all cognitive measures involved at least four subjects, the 31 M time point for several measures had data from only two subjects, so that time point was omitted from the graphing/analysis for those measures. All data points are graphed as means ± SEM, with statistically significant effects requiring a “*p*” value of 0.05 or a greater level of significance.

## 3. Results

### 3.1. Long-Term Safety of TEMT and TEMT’s Effects on Brain Regional Volumes

Over this 31-month study, most subjects received around 600 one-hour in-home treatments, as provided by their caregiver. As evidenced by diary records kept by the caregivers, no subject exhibited any recurrent changes in anxiety level/mood, eating/drinking, or daily movement activities. In addition, subjects did not complain of brain sensations, headaches, or any other side effects of TEMT during or following treatment. Caregiver recordings of blood pressure and temperature before, during, and 30 min after treatments also did not show consistent changes in these physiologic parameters linked to TEMT administration. The Adverse Event Assessment performed during every clinical visit indicated no adverse events of treatment, and the clinical assessment of suicidal tendencies revealed no suicidal tendencies resulting from treatment.

In support of their diagnosis of AD, pre-treatment (baseline) MRI scans (SWI/FLAIR sequences) from all five subjects of this study indicated that they had significant frontal/parietal lobe atrophy, hippocampal/temporal lobe atrophy, and/or global (diffuse) cerebral cortical atrophy (Table 1). Follow-up MRI scans taken at 19 months and/or 31 months from baseline for four of the five subjects revealed no visible induction of tumors or brain microhemorrhages over the period of treatment/no treatment. Moreover, there was typically no progression in hippocampal/temporal lobe atrophy or global cerebral atrophy in qualitative comparisons of MRI scans from the 19-month and/or 31-month time points compared to baseline. One subject did, however, have a qualitatively assessed decrease in temporal lobe/hippocampal volume between baseline and 19 M, with no further decrease between 19 M and 31 M. In addition, there were no qualitative increases in lateral ventricular volume over this same lengthy period. These MRI results indicate no deleterious TEMT-induced brain abnormalities and, generally, no decreases in global or region brain volumes over a 2–2½-year period.

### 3.2. Long-Term TEMT Effects on Cognition/Functional Measures in AD Subjects over a 2½-Year Period

Five mild/moderate AD subjects were evaluated in a comprehensive array of eight cognitive/functional measures within six established tasks at baseline, 2, 10, 12, 14, 19, 21, 24, 27, and/or 31 M—a 2½-year period during which no treatment was given between 2 and 10 M and between 14 and 19 M after baseline (see Figure 2 for the timeline). Thus, daily TEMT was given for 18 months within a 31 M study period (i.e., 58% of the study period).

Figure 3 shows the effects of long-term TEMT on overall ADAS-cog13 performance (a) and on a the primary sub-measure of that task, immediate recall (b). Because the present study did not involve a control/placebo treatment group, a longitudinal assessment of ADAS-cog overall scores over a 24-month period is also shown for a large group (*n* = 90 at baseline) of untreated mild AD subjects in an earlier study [34] for comparison. Demographics and baseline ADAS-cog scores from this earlier study were very similar to those of the present study. The 4–5 point yearly decline in ADAS-cog scores reported in this earlier study is typical for mild/moderate AD subjects. By contrast, an immediate improvement of around 3 points was seen following the initial 2 months of TEMT period. Thereafter, and following an 8 M period of no treatment, TEMT-treated AD subjects continued to show no decline compared to baseline in ADAS-cog13 through 12 M and were largely stable in performance from 19 M through 27 M of TEMT. Comparing ADAS-cog13 performance across time through 27 M using a multivariate mixed-effects model revealed no significant decline in ADAS-cog13 overall through the 27 M time point (Table 2). In addition, there was no significant change in ADAS-cog13 overall in a direct comparison of the 27 M time point (35.5 ± 1.9) vs. baseline (29.3 ± 4.1) (Table 2). Nonetheless, a declining performance from 10 M through 27 M was evident, with increased variability (larger SEMs) between subjects at and beyond 19 M. In the “Immediate Recall” component of ADAS-cog (Figure 3b), the number of words recalled at baseline was maintained throughout 27 M of testing, as evidenced by no significant change in the trajectory across that lengthy period in a mixed-effects model and no significant difference in the number of words recalled for baseline vs. 27 M (Table 2).

**Figure 4:**
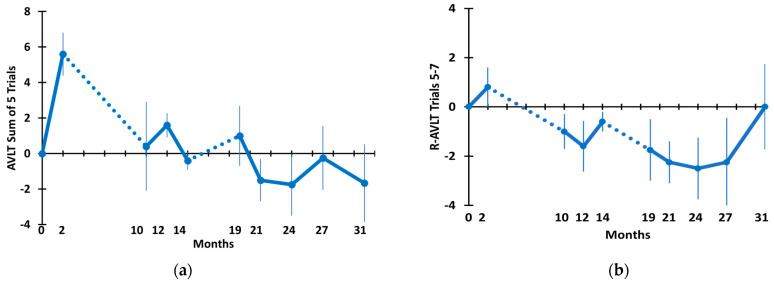
(**a**) Rey Audio Verbal Learning Test sum of five trials and (**b**) Rey AVLT retroactive interference through 31 months. Dotted lines indicate the 8 M and 5 M periods of no treatment.

Figure 4 depicts TEMT’s effects on Rey AVLT sum of five trials (a) and retroactive interference (b). For both measures, an initial increase in performance over the first 2 months of daily treatment was followed by a decline immediately following an 8-month period of no treatment. For the AVLT sum of five trials, performance was stabilized at baseline levels from the 10 M time point through the end of testing at 31 M. The overall stability in AVLT sum of five trials scores across this lengthy period is demonstrated by no change over time compared to baseline in a mixed-effects model as well as no difference when comparing the final testing at 31 M to the baseline performance (Table 2). The trajectory for change in retroactive interference was somewhat more complex, with a general downward trajectory but a recovery to baseline levels by the end of testing. The downward trajectory through most of the 31 M period explains why a nearly significant (*p* = 0.051) decrease in retroactive interference occurred over time in a mixed-effects model (Table 2). However, because of recovery by 31 M back to baseline levels, there was no difference between the baseline and 31 M performances on this measure (Table 2).

**Figure 5:**
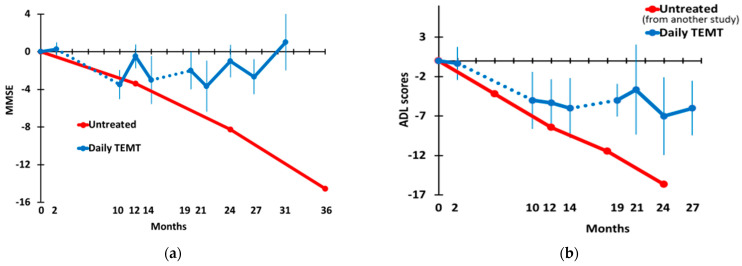
(**a**) Mini-Mental State Exam (MMSE) and (**b**) Activities of Daily Living (ADL). Dotted lines indicate the 8 M and 5 M periods of no treatment. For both the MMSE and ADL graphs, the red data points are from earlier clinical studies evaluating these two measures longitudinally in untreated mild/moderate AD subjects. SEMs were not available from either earlier study.

MMSE and ADL scores in TEMT-treated AD subjects through 31 M are shown in Figure 5a,b, respectively. MMSE scores dropped during the initial 8 M period of no treatment but stabilized and improved back to baseline levels thereafter from the 10 M through the 31 M time point. For comparison, the longitudinal MMSE scores from untreated mild/moderate AD subjects in an earlier study [35] through 36 M are graphed in red (*n* = 106; mean MMSE score at baseline = 21.1). The demographics and baseline MMSE scores from this earlier study were very similar to those of the present study. The initial decline in MMSE scores from TEMT-treated AD subjects after the 2 M time point paralleled the decline in scores from untreated AD subjects. However, TEMT-treated subjects stabilized/improved beginning at 10 M, while untreated AD subjects continued a precipitous decline in MMSE scores, averaging around 4–5 points per year [35]. For TEMT subjects, evaluating the change in MMSE scores across 31 M in a mixed-effects model revealed no change, as did a direct comparison of MMSE scores at 31 M vs. baseline (Table 2).

The profile of ADL scores in TEMT-treated AD subjects paralleled that of MMSE scores over a 27 M period (Figure 5b). Specifically, a drop in ADL scores during the 8 M period of no treatment was followed by the stabilization of ADL scores with the re-initiation of treatment at 10 M. Longitudinal ADL scores from untreated mild/moderate AD subjects in an earlier study [36] are shown for comparison in Figure 5b (*n* = 89; mean MMSE score at baseline = 19.0). The demographics and baseline ADL scores from this earlier study were comparable to those of the present study. The stabilization in ADL scores exhibited by TEMT-treated AD subjects beginning at 10 M is in sharp contrast to the 4-point declines at six-month intervals through 24M shown by untreated AD subjects [36]. This stabilization was statistically verified by both no change over time (mixed-effects model) and a direct comparison of baseline ADL scores vs. 27 M scores (Table 2).

**Figure 6:**
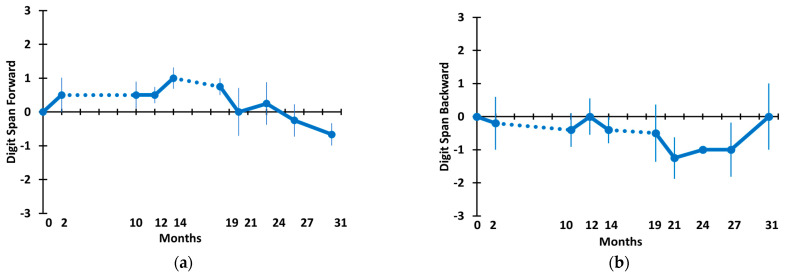
(**a**) Digit Span Forward and (**b**) Digit Span Backward through 31 months. Dotted lines indicate the 8 M and 5 M periods of no treatment.

Figure 6 shows the Digit Span Forward (a) and Digit Span Backward (b) scores for AD subjects through the 31 M TEMT period. For both measures, only small one-word fluctuations occurred in means throughout this long-term period. For both the change-over-time analysis (mixed-effects model) and the direct comparison of scores at 31 M vs. baseline, there were no significant differences for Digit Span Forward and Backward (Table 2).

**Figure 7:**
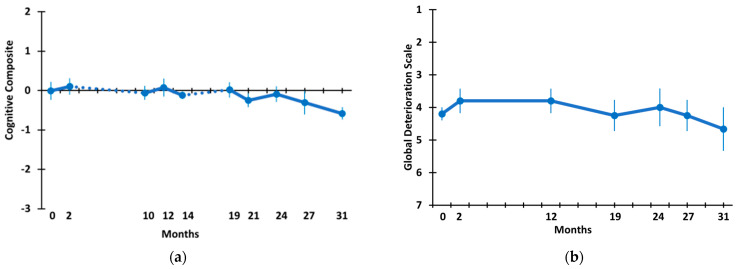
(**a**) Cognitive Composite of the eight measures in Figure 3, Figure 4, Figure 5 and Figure 6 and (**b**) the Global Deterioration Score (GDS). Dotted lines in (**a**) indicate the 8 M and 5 M periods of no treatment.

A “Cognitive Composite” of overall cognitive performance for the five AD subjects given TEMT was created, utilizing scores from all eight measures in the six tasks presented in Figure 3, Figure 4, Figure 5 and Figure 6. These cognitive composite results are graphed in Figure 7a and show no change, with little fluctuation in the overall cognitive performance across the 31 M period. This was statistically verified by the change-over-time mixed-effects analysis (Table 2) as well as a direct comparison of the cognitive composite scores at 31 M vs. baseline (−0.01 ± 0.23 vs. −0.58 ± 0.16; *p* = 0.328, t = 1.28).

A final cognitive assessment involved the caregiver ratings of their AD patients during clinical visits on the 7-stage Global Deterioration Scale (GDS). GDS stage 1 indicates no cognitive impairment, and stage 7 indicates very severe cognitive impairment. As shown in Figure 7b, the GDS scores remained basically unchanged during the 31 M period of TEMT, with patients starting baseline with an average GDS score just above stage 4 (moderate cognitive impairment) and finishing the 31 M period with no significant change in the caregiver GDS rating (4.20 ± 0.20 vs. 4.67 ± 0.67; *p* = 0.423, t = −1.00).

### 3.3. Long-Term Effects of TEMT on AD Markers in Both CSF and Plasma over a 2+ Year Period

As indicated in Figure 2, there was an 8-month period of no treatment between 2 M and 10 M into TEMT, so a total of 6 months of daily TEMT was provided during the initial 14-month period of TEMT. For the CSF analyses, baseline samples were compared to the final CSF samples taken at 14 months from baseline or to CSF samples taken at 2 M, 10 M, and 14 M from baseline. For the plasma analyses, baseline samples were compared to either 14 M or 27 M plasma samples.

**Figure 8:**
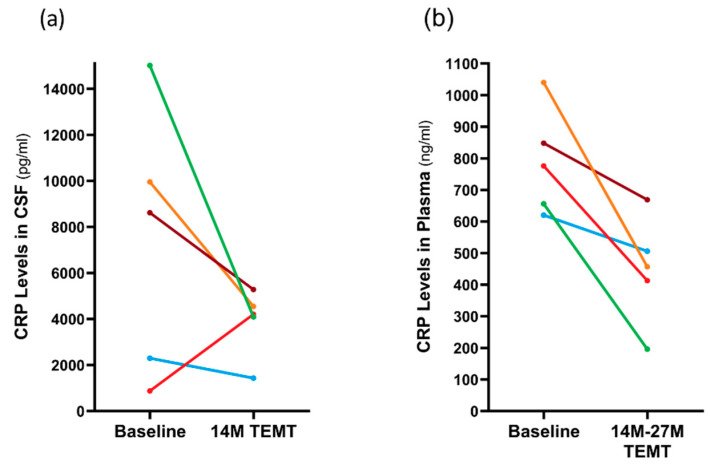
For individual subjects, effects of 14 M TEMT on C-reactive protein (CRP) levels in CSF (**a**) and effects of 14–27 M of TEMT on plasma CRP levels (**b**). Each color depicts the same subject.

C-reactive protein (CRP) is a marker of inflammation in both the brain/CSF and body/plasma. In CSF, the 14 M period of TEMT reduced CRP levels in four of the five subjects compared to baseline by an average of 51% (Figure 8a). The lone subject that showed a TEMT-induced increase at 14 M was the one non-performer in several tasks (see Section 2.12). In plasma, all five subjects showed a significant TEMT-induced decrease in CRP levels at 14 M or 27 M into treatment (−43%; *p* = 0.017) compared to baseline levels (448 ± 76 ng/mL vs. 788 ± 75 ng/mL), as depicted in Figure 8b.

**Figure 9:**
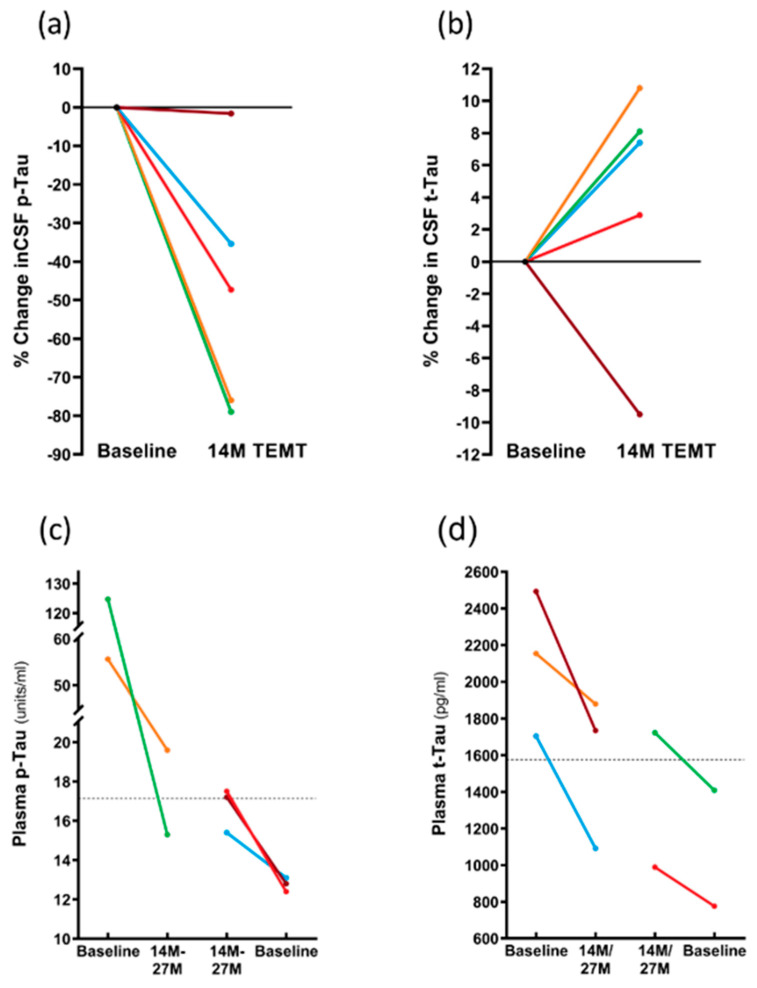
(**a**,**b**) For individual subjects, effects of 14 M TEMT on percent change in CSF levels of p-tau217 (**a**) and t-tau. (**c**,**d**) Effects of 14–27 M TEMT on plasma levels of p-tau217 (**c**) and t-tau (**d**) for individual subjects. Each color represents results from the same subject.

As was the case for the CRP levels in CSF, the p-tau217 levels in CSF were also reduced at 14 M by TEMT, with reductions of 35–79% evident in four of the five subjects (Figure 9a). In these four subjects, the overall comparison of p-tau217 levels in the CSF at 14 M (7.2 ± 3.7 units/mL) compared to baseline levels (16.8 ± 5.3 units/mL) revealed a significant TEMT-induced decrease of 57% (*p* = 0.031). In contrast, TEMT induced increases in the CSF levels of total tau (t-tau) in the same four subjects at 14 M that were nearly significant (315 ± 40 vs. 347 ± 36; *p* = 0.059) (Figure 9b).

The effects of TEMT on plasma p-tau217 and total tau levels were dependent on the baseline levels in individual subjects. Specifically, if the baseline levels of plasma p-tau217 or total tau were higher, TEMT reduced those levels at 14 M or 27 M (Figure 9c,d). Conversely, if baseline levels of plasma p-tau217 or total tau were lower, TEMT increased those levels at 14 M or 27 M (Figure 9c,d). For example, subjects with higher baseline plasma total tau levels experienced an overall 26% TEMT-induced reduction, while those with lower baseline levels experienced an overall 25% TEMT-induced increase. Thus, a modulation or “rebalancing” toward a convergent level for both plasma p-tau217 and total tau levels was induced by long-term TEMT. For plasma Aβ1-40 and Aβ1-42 levels, there was also evidence of a modulation/rebalancing effect of TEMT, although the overall plasma levels of both were too low to validate this effect (data not presented).

**Figure 10:**
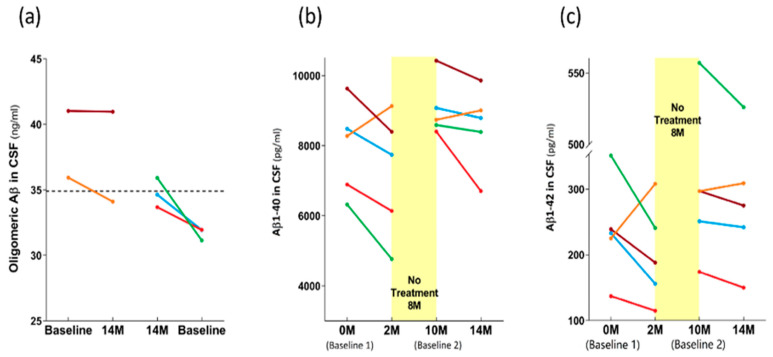
For individual subjects, effects of 14 M TEMT on CSF levels of oligomeric Aβ (**a**), Aβ1-40 (**b**), and Aβ1-42 (**c**). Each color represents results from the same subject.

As was the case for the plasma levels of both p-tau217 and total tau (Figure 9c,d), the effects of TEMT on the CSF oligomeric Aβ levels were dependent on the baseline levels in individual AD subjects. Specifically, if the baseline CSF levels of oligomeric Aβ were lower, TEMT increased those levels at 14 M and vice versa if baseline levels were higher (Figure 10a).

The effects of TEMT on Aβ1-40 and Aβ1-42 in CSF are best visualized by evaluating the levels at all four CSF collection points: 0 months (baseline 1), 2 M, 10 M (baseline 2), and 14 M, with an 8-month period of no treatment between the 2 M and 10 M collections. For four of the five AD subjects, the 2 M and 4M periods of daily TEMT resulted in CSF reductions in both Aβ1-40 and Aβ1-42 compared to their respective baseline levels (Figure 10b,c). For these subjects, baseline 1 vs. the initial 2 M of TEMT revealed significant reductions in CSF levels of both Aβ1-40 (7830 ± 754 vs. 6759 ± 816 pg/mL; *p* = 0.012) and Aβ1-42 (238 ± 44 vs. 175 ± 27 pg/mL; *p* = 0.042). Comparing baseline 2 levels to those after 4M of TEMT (i.e., between 10 M and 14 M) showed a significant reduction in CSF levels of Aβ1-42 (320 ± 83 vs. 298 ± 80 pg/mL; *p* = 0.018), although this comparison for Aβ1-40 in CSF did not reach significance (9123 ± 457 vs. 8436 ± 655 pg/mL; *p* = 0.141). For both Aβ1-40 and Aβ1-42 in CSF, all subjects exhibited increased CSF levels after the 8 M period of no treatment compared to the end of the initial 2 M treatment period (Figure 10b,c).

## 4. Discussion

There are presently no published papers showing that any therapeutic intervention can stabilize/stop or reverse the progressive cognitive decline in AD, especially over an extended period of time. Indeed, the main goal of pharmaceutical companies now appears to be to slow down the AD cognitive decline rather than stopping or reversing it [3]. New nonpharmaceutical approaches against AD are now clearly warranted, such as the bioengineering-based technology we have developed called transcranial electromagnetic treatment (TEMT). We have previously reported that daily TEMT over a 2-month period reversed cognitive impairment, provided fMRI brain imaging benefits, and induced predictable changes in CSF/blood AD markers [27]. The present study greatly extends this initial study by providing TEMT through 31 months from the initial study’s baseline in the same small group of mild/moderate AD subjects. We report the safety and apparent widespread stabilization/arrest of cognitive decline in those TEMT subjects across eight measures in six tasks through this extended 31-month (2½-year) period. Moreover, TEMT induced decreases, increases, or modulation/rebalancing effects on various AD markers in both CSF and plasma. Nonetheless, the present results should be viewed with caution until larger, controlled clinical studies are performed with TEMT in AD subjects.

### 4.1. Safety of TEMT

A primary goal of this 31-month study was to determine the safety of daily, long-term, global TEMT. In our initial 2-month pilot study (1st protocol of the present study), no adverse side effects of the treatment were noted over this period involving a total of 120 one-hour treatments administered twice daily [27]. This safety profile of TEMT continued and is underscored by the present study, wherein most subjects received around 600 one-hour treatments over a period of approximately 2½ years. Specifically, no adverse effects were reported by the subjects or caregivers at clinical visits, wherein Adverse Effects Assessments and suicidal tendencies were recorded. In addition, the diaries kept by caregivers indicated no repeated changes in daily activities or anxiety level/mood and no consistent changes in blood pressure or body temperature linked to TEMT. Indeed, there are many epidemiologic studies that collectively find that humans exposed to EMF frequencies/power levels similar to those of the present study (via mobile phones) display no adverse behavioral or physiologic side effects or any provocation of brain cancer [37,38].

A comparison of the anatomic brain MRI scans taken at baseline to those taken at 19 and/or 31 months indicated no visible induction of tumors, micro-hemorrhages, or the development of any other brain abnormalities during long-term TEMT. With the exception of one subject between the baseline and 19 M time point, no progression in hippocampal, temporal lobe, or global cerebral atrophy was observed in these MRI scans compared to baseline as well as no increase in the lateral ventricular volume. Although a follow-up quantitative analysis is needed for definitive assessment, these qualitative MRI results suggest that TEMT arrested or slowed the brain atrophy that occurs progressively in AD. Along that line, longitudinal MRI studies in AD subjects have reported cerebral atrophy occurring, particularly in the hippocampal/temporal lobe, over intervals as short as one year between MRI scans [39,40].

All of the aforementioned endpoints jointly indicate that global brain TEMT administration at the currently used parameters was entirely safe in AD subjects, with no deleterious side effects over a lengthy 31-month study period, and provide qualitative evidence of perhaps even stopping or slowing brain atrophy during that period.

### 4.2. Long-Term Cognitive Benefits of TEMT

Over an extended period of 31 M (2½ years), the present study reports an extensive stabilization/arrest of cognitive decline in mild/moderate AD subjects that received TEMT across eight measures in six tasks. Although the number of AD subjects was small, the SEMs were generally small or modest in size, indicating generally limited variability in performance among subjects. Compared to baseline, not a single significant difference was seen in the eight measures evaluated separately or collectively when all eight measures were combined into an overall composite cognitive score. Nonetheless, it could be argued that the small number of subjects, and limited statistical power therein, contributed to no statistical differences being determined across time or in a direct comparison of baseline vs. 27–31 M performance in the eight measures. Countering this premise, first, were the caregiver assessments of the GDS stage of AD cognitive impairment of their loved one, wherein there was no change in GDS scores during the 31 M treatment period compared to baseline. Second, there was only one measure out of 19 where a decline in cognitive performance was even close to being significant (see Table 2). Third, two earlier clinical studies involving electromagnetic approaches very different from the present study’s TEMT also reported cognitive benefits to AD subjects following 4–5 weeks of treatment [41,42].

It should be underscored that the aforementioned cognitive benefits were present with treatment only 58% of the time (18 of 31 months). This entreats the question of how cognitive performance would have been affected if continuous (daily) TEMT had been administered for the entire 31 M period without two breaks of 8 and 5 months. Our upcoming phase IIb/phase III (pivotal) clinical trial will involve continuous (daily) TEMT administration to address this important question. With multiple cognitive tests during the 31 month period, the possibility of practice effects (repeated testing effects) should also be addressed. In that regard, cognitive testing involving the Uniform Data Set (UDS), which was incorporated into all Alzheimer’s Disease Research Centers in 2005, indicated that while both cognitively normal and mild cognitive impairment subjects showed significant practice effects on repeated cognitive testing, no significant practice effects were observed in AD subjects [43].

Although many Alzheimer’s transgenic mouse studies over the past 20 years have reported cognitive benefits of numerous pharmacologic agents against the cognitive impairment they exhibit, none of these preclinical mouse studies appear to have translated to similar benefits in AD patients. Along this line, our own preclinical studies involving AD transgenic mice have consistently demonstrated the ability of TEMT to prevent and reverse cognitive impairment [23,24,25]. However, unlike most other therapeutic interventions against AD, TEMT’s cognitive benefit in our preclinical studies translated to a clinical benefit to AD subjects. Aside from the ability of TEMT technology to target multiple aspects of the AD process (see Section 4.4), this successful translation of mouse work to the clinical realm appears to be due to a fortuitous selection of TEMT treatment parameters (e.g., EMF frequency, EMF power level, and daily treatment paradigm), treatment parameters that were shown to be safe and efficacious in our preclinical mouse studies that were then translated into our AD clinical studies.

*ADAS-cog.* The ADAS-cog battery of cognitive measures has long been a benchmark for evaluating cognitive treatment effects in AD clinical studies, particularly with the newest version, consisting of 13 cognitive measures (ADAS-cog13), that was used in the present study. The lack of statistical decline in ADAS-cog scores, both over time and comparing 27 M to baseline scores, suggests that TEMT had greatly slowed or stopped the ADAS-cog decline over that period. This was despite receiving treatment for only a little over half of the months during this time period. The lack of any decline in ADAS-cog over the first year after baseline is in contrast to the typical decline of 4–5 points for mild/moderate AD subjects over that same period [34]. This approximate 12-month treatment benefit continued for TEMT subjects from the 12 M through the 27 M time points since there was always around a 12-month differential between the ADAS-cog scores horizontally comparing treated AD subjects to untreated AD subjects from an earlier study [34]. However, there was a general trend for declining performance from the above-baseline levels at 10 M through 27 M. Among the sub-measures of the ADAS-cog13, one of the more important ones is immediate recall, which was clearly maintained over the 27 month period in AD subjects that received TEMT. This is in contrast to untreated AD subjects, where decreases in ADAS-cog immediate recall are seen across a similar 2-year period of time [44].

*Rey AVLT and Digits Forward and Backward.* Aside from ADAS-cog, a second task involving multiple measures that is often utilized to evaluate cognition in AD studies is the Rey Audio-Verbal Learning Test (AVLT). One of its two primary measures, the sum of five trials, is analogous to the immediate recall measure of ADAS-cog and similarly showed no decline over a 31 M period involving TEMT. Rey AVLT’s other primary measure, retroactive interference, is important because requiring subjects to name items from an initial list after they have been asked to name items from a second list (cognitive interference) is very sensitive to cognitive decline. Indeed, cognitive interference is a measure that clearly discriminates AD subjects from those with MCI and non-cognitively impaired aged individuals [45]. For the Digits Forward and Digits Backward tasks, the stable cognitive performance through 31 M shown by AD subjects given TEMT again underscored the apparent ability of this therapeutic intervention to stop/stabilize cognitive decline in AD subjects over a lengthy period of time.

*MMSE and ADL.* The TEMT-induced effects on MMSE “cognitive” performance and ADL “functional” performance were similar over a 27–31 month treatment period. Specifically, the performance in both tasks declined during the initial 8-month period of no treatment but then either stabilized or improved back to baseline levels with the re-initiation of TEMT. Although the performance in both of these tasks was sensitive to this lengthy period of no treatment, the second 5-month period of no treatment did not result in decreased performance, suggesting that long-term TEMT may stabilize these two measures, even with a lengthy period between daily treatments. The stabilization/improvement provided by TEMT in these two tasks is further underscored by the rather rapid decline in both tasks that is expected without TEMT, as exemplified by a comparison with untreated mild/moderate AD subjects from earlier studies [34,35]. The ADL measure is particularly important as a “functional” activity measure provided by the caregiver in assessing the AD subjects’ ability to perform various daily activities in the home environment (e.g., grooming, reading, and conversation). Inasmuch as the FDA currently desires any therapeutic they consider for approval against AD to have both cognitive and functional activity benefits, the ability of TEMT to stabilize ADL is important. Nonetheless, the verification of these ADL effects of TEMT will require larger, controlled clinical studies, as is the case for all of this study’s cognitive results.

*Cognitive Composite.* A “Cognitive Composite” score involving multiple combined measures would inherently be advantageous in demonstrating (or not) a widespread cognitive-enhancing ability of any given AD therapeutic. Although such cognitive composite scores have been used occasionally to track “preclinical” AD cognitive decline [46,47,48], they are rarely used in AD intervention trials [49,50]. Even in these few AD intervention trials, the cognitive composite is limited to only two measures (ADAS-cog and ADL) in what is called an Integrated Alzheimer’s Disease Rating Scale (iADRS). By contrast, the present study’s cognitive composite involved eight measures taken from six tasks and calculated at multiple time points through 31 months of TEMT. In so doing, a remarkable stability/maintenance of “overall” cognitive performance over multiple domains was demonstrated, both over time and when comparing the final (31 M) composite scores to those at baseline. To our knowledge, this is the first AD therapeutic study assessing composite scores from more than two measures, much less attaining a composite stability/stoppage of progressive AD cognitive decline over a protracted 2½-year period.

### 4.3. Long-Term Effects of TEMT on AD Markers in Blood and CSF

Long-term TEMT through 14–27 months induced changes in, or the modulation of, major AD markers in both the CSF and plasma. In the CSF, long-term TEMT induced reductions in the levels of C-reactive protein, p-tau217, Aβ1-40, and Aβ1-42 while modulating CSF oligomeric Aβ levels. In the plasma, long-term TEMT modulated/rebalanced the levels of both p-tau217 and total tau. These TEMT-induced effects on AD markers were evident many months after the start of treatment and may have contributed to the presently reported stoppage of cognitive/functional decline over a 2½-year period in the same AD subjects.

C-reactive protein (CRP) is a marker of inflammation that most studies have reported to be increased in plasma from AD subjects [51,52] as well as being strongly associated with the neurodegenerative inflammatory processes in AD brains [53]. Along this line, AD is often portrayed as a disease of the brain and/or peripheral inflammation involving an “over-activated” immune system, as evidenced by elevated cytokine levels in the brain [54] and blood [55,56,57]. Indeed, many AD researchers believe that AD involves, at least partially, a low-grade inflammation in the brain or periphery [58]. In the present study, long-term TEMT spanning 14–27 months universally reduced CRP in plasma by an average of 43% and by an average of 51% in CSF for four of the five AD subjects. Thus, TEMT may have reduced the overall inflammation in both the brain/CSF and the periphery/plasma over an extended 2+ year period. This is consistent with our recent report involving the same AD subjects [59] that showed that the initial 2 M period of TEMT modulates or “rebalances” 11 of 12 cytokines in the brain and/or plasma. Thus, the largely proinflammatory immune status of AD subjects would appear to have been “re-balanced” to a less inflammatory state by TEMT, as evidenced by reduced CRP levels in both CSF and plasma. This reduced inflammation alone could be an important factor in the long-term cognitive stability presently reported with TEMT.

Long-term TEMT impacted the CSF levels of both p-tau217 and total tau (t-tau) in AD subjects. At 14 months into treatment, the p-tau217 levels in CSF were significantly decreased, while the CSF levels of t-tau were increased. These opposite tau effects of TEMT in CSF from AD subjects are exactly what would be expected based on our recent finding that the AD brain homogenates exposed to an identical TEMT treatment protocol over seven days responded with (1) a decrease in p-tau levels (i.e., a reversal of abnormal tau hyper-phosphorylation) and (2) an increase in t-tau levels (which is almost exclusively monomeric) [ref.-unpublished observations]. Since the in vitro dephosphorylation of p-tau with protein phosphatase 2A inhibits tau oligomerization [60], TEMT’s ability to dephosphorylate p-tau accomplishes this same action, presumably preventing or disaggregating p-tau oligomer formation in the brains of this study’s AD subjects. The currently reported decrease in the CSF levels of p-tau by TEMT is particularly noteworthy in view of studies showing that high CSF levels of p-tau217 are an accurate biomarker for AD [61]. In addition, a significant correlation exists between CSF p-tau levels and cognitive dysfunction in AD subjects [62]. TEMT’s ability to substantially decrease the CSF levels of p-tau217 would then suggest that this is yet another contributing factor to the TEMT-induced stoppage of cognitive decline demonstrated in the present study.

In contrast to the CSF effects of TEMT to decrease p-tau217 and increase t-tau, the long-term effects of TEMT on the “plasma” levels of these two AD markers were modulatory or “rebalancing” in nature after a 14 M or 27 M period of treatment. Specifically, if the baseline p-tau217 or t-tau levels were higher in individual AD subjects, TEMT reduced those levels while inducing just the opposite effect (i.e., an increase) if baseline levels were lower. This TEMT-induced modulation to convergent levels was observed for nine plasma cytokines in the same subjects after their first 2 months of TEMT treatment, as we recently reported [59]. Both results strongly suggest that the same TEMT-induced modulatory mechanism is responsible for regulating the plasma levels of p-tau217 and that t-tau is regulating the levels of many plasma cytokines. The determination of exactly what this plasma regulatory mechanism may be and how TEMT is activating it is the subject of a current investigation.

For four of the five AD subjects, the CSF levels of both Aβ1-40 and Aβ1-42 were reduced after an initial 2 M of TEMT as well as after an ensuing 4M period of TEMT (with an 8 M period of no treatment interposed). In AD subjects, the CSF levels of Aβ1-42 are consistently decreased compared to aged controls [31,63], although the Aβ1-40 levels in CSF appear to be increased in AD [64]. As such, any interpretation of the presently observed reductions in both Aβ isoforms following both TEMT periods should be limited. This is particularly the case in view of our earlier reported small increases in both Aβ42 and Aβ40 within the CSF following the initial 2 M of TEMT period, albeit with a greater number of AD subjects [27]. Nonetheless, the fact that the CSF levels of both Aβ isoforms increased in these four subjects after the 8 M period of no treatment suggests that the TEMT-induced decreases in CSF levels of Aβ had some meaningful affect. What can further be established is that, by the inclusion of soluble

Aβ1-40 and Aβ1-42 levels in the CSF, TEMT has affected most major AD markers in the CSF and/or plasma (e.g., p-tau, t-tau, oligomeric Aβ, CRP, and cytokines), an attribute that few, if any, AD therapeutics in clinical trials can claim.

### 4.4. Mechanisms of TEMT Action

The main mechanism of TEMT action responsible for its cognitive stabilization of AD appears to be a disaggregation of toxic intraneuronal Aβ and tau oligomers. Numerous recent studies have linked these soluble aggregates to the initiation and development of AD—not the large, insoluble Aβ deposits in extraneuronal neuritic plaques or the insoluble phospho-tau deposits that comprise intraneuronal neurofibrillary tangles [4,5,6,7,8,9,10,11,12,13,14,15,16,17,18,19,20,21]. We and others have published studies involving AD transgenic mice consistently showing the ability of TEMT to prevent Aβ aggregation and to disaggregate both soluble and insoluble Aβ aggregates in their brains [13,23,24,65,66]. Our latest studies used human AD brain homogenates to demonstrate TEMT’s ability not only to disaggregate oligomeric Aβ but also to disaggregate oligomeric tau in such AD homogenates [26]. This may be critical because Aβ and tau oligomers can independently cause neuronal dysfunction, and tau may be the intermediary for driving Aβ-induced synaptotoxicity [11].

There is considerable evidence that the exact mechanism through which TEMT induces the disaggregation of Aβ and tau oligomers/aggregates involves the destabilization or weakening of H-bonds between individual oligomer monomers, resulting in their eventual breakage. Such destabilization occurs through reduced dipole–dipole interactions (dielectric loss), vibration, and/or resonance phenomena [67,68,69,70]. In that regard, the “monomeric” β-sheet structure of Aβ and tau proteins are held together into oligomers by H-bonds that are at an abnormal angle and longer, making them easier to disrupt by TEMT. Specifically, the average H-bond length in a typical protein’s secondary structure is 2.5–3.0 Å [71]. However, the H-bond length between individual Aβ monomers in Aβ oligomers is 4.7–4.8 Å, indicating that they are very weak H-bonds and are much more susceptible to breaking [72].

In addition to toxic protein disaggregation, we have also reported that TEMT induces mitochondrial enhancement in AD transgenic mice through (1) the removal of toxic intra-mitochondrial Aβ oligomers and (2) the direct activation of complex IV [13]. Most recently, we have identified an additional mechanism of TEMT action, namely, a regulation/rebalancing of the brain and body’s cytokine system [59]. This newest mechanism is important because AD is consistently linked to an imbalance of pro- and anti-inflammatory cytokines in the brain and/or periphery [59]. Unlike pharmacologic interventions against AD, TEMT can penetrate the blood–brain barrier and easily enter the brain’s neurons/glial cells to induce toxic protein disaggregation, mitochondrial enhancement, and immune regulation. In fact, this multimechanistic capability of TEMT appears to provide a combination of AD therapeutic actions that other therapeutic interventions against the disease have thus far been unable to attain. Such a multi-targeting therapeutic probably affords the best opportunity to stabilize or reverse the progressive cognitive impairment of AD.

In addition to AD, there are multiple other neurologic disorders where TEMT’s multiple mechanisms of action could provide far-reaching therapeutic benefits. These disorders include Parkinson’s disease [73,74], Lewy body dementia [75,76], and frontotemporal lobe dementia [77,78], all of which are characterized by toxic protein aggregation in the brain, mitochondrial dysfunction in the brain, and/or a dysregulation/imbalance of the cytokine (immune) system in the brain or vasculature. Thus, TEMT does not simply represent a horizontal step involving an existing technology but rather represents a “vertical” step to an entirely new technology for neuromodulation.

### 4.5. Other Neuromodulatory Approaches against AD and Their Mechanisms of Action

In addition to TEMT, there are a number of other neuromodulatory approaches against AD that have been evaluated clinically. Chief among them are transcranial magnetic stimulation (tMS) and transcranial direct current stimulation (tDCS). In contrast to the interdigitated electric and magnetic waves generated by TEMT, tMS and tDCS use magnetic waves or electric current, respectively, to generate electric fields in the cerebral cortex that can enhance the excitability/activity of neurons or to modulate cortical brain oscillations [79,80]. Neuronal oscillatory activity, which is thought to orchestrate brain function/dysfunction, is abnormal in AD subjects [79,81]. Since oscillatory activity is modifiable by tMS and tDCS, such neuromodulatory actions may be able to normalize brain oscillations as a therapeutic intervention against AD [79]. However, tMS or tDCS studies in AD subjects have yet to show a restoration of normal oscillatory activity concurrent with cognitive benefits in AD subjects. Numerous small and modestly sized studies providing tMS or tDCS to AD subjects have been reported to improve cognition, as measured by ADAS-cog or MMSE [80]. However, the largest tMS clinical study to date found no cognitive benefit immediately following 6 weeks of tMS in AD subjects [82]. In addition, tMS or tDCS studies in AD subjects almost universally have not measured AD markers and have not involved treatment for more than 6 weeks. Nonetheless, clinical trials of tMS and tDCS in AD subjects continue to be warranted and are still ongoing.

Similar to the findings with tMS and tDCS, we have previously reported that TEMT also induces increased neuronal activity in both aged AD transgenic and aged normal mice [25,83]. Relatedly, some AD patients in our initial 2-month clinical trial showed enhanced neuronal activity, as determined by FDG-PET brain scanning before and after treatment [27]. Since a progressive decline in neuronal activity begins well before the diagnosis of AD [84,85], early intervention with therapeutics that increase neuronal activity (e.g., TEMT, tMS, and tDCS) could act to stabilize or improve cognitive function through neurophysiologically induced changes in neuroplasticity/brain oscillations. Thus, TEMT may share some neuronal enhancement/neuroplasticity actions with both tMS and tDCS that could be contributing to the cognitive benefits presently being reported. Unfortunately, clinical studies investigating TEMT’s modulation of neuronal oscillations have not yet been conducted.

### 4.6. Study Limitations

There are some limitations to this long-term TEMT study that should be noted. First, as with most pilot studies, all subjects received treatment, so there was no untreated/placebo group to compare the cognitive performance or blood/CSF AD markers to for definitively establishing a treatment effect. Nonetheless, the fact that there was no significant decrease in cognitive performance over six AD tasks and eight measures therein, no decrease in “overall” composite cognitive performance, and no change in GDS scores strongly suggests that TEMT stabilized cognitive performance through a 2½-year period. Moreover, it is difficult to disregard the CSF and plasma changes in AD markers provided by TEMT. Nevertheless, a definitive determination of TEMT’s cognitive/AD marker benefits against AD will require controlled clinical trials.

A second limitation encompasses the relatively small number of AD subjects (initially eight) in this long-term study. It should be mentioned that initial studies involving neuromodulatory approaches against AD, such as tMS [41,42], deep brain stimulation [86], and light therapy [87,88]), have typically involved 10 or less subjects. Nonetheless, the small number of AD subjects involved may have statistically contributed to the lack of cognitive decline in some of the eight cognitive measures evaluated. It is important to underscore that this study involved many endpoints in a diversity of cognitive measures and AD markers over a long 2½-year period. In addition, the comprehensive three-protocol design of this study involved active participation by caregivers (around 600 one-hour treatments) and clinical/research staff. In this long-term study, the presence of TEMT-induced cognitive/functional maintenance and changes in multiple AD markers in both the CSF and plasma provide a firm foundation for the controlled phase IIb/III clinical trial that has been planned.

A final limitation of this study involves the brain penetration of TEMT. Our FDTD (ANSYS) computer simulations show that, at the frequency (915 MHz) and power level (1.6 W/kg) utilized in this study, TEMT penetrates considerably into the subcortical regions (Figure 1c). However, a higher power level would be desirable because it would likely provide more a robust treatment to deep brain regions such as the hippocampus, resulting in greater cognitive benefits. In that regard, we have developed a second-generation (Gen2) MemorEM head device that provides the option of a power level above 1.6 W/kg. This Gen2 device will be used in all of our future clinical trials involving TEMT.

## 5. Conclusions

Despite many clinical trials over the past 20 years, there are presently no published papers showing the ability of any therapeutic intervention to stabilize/stop the progressive cognitive decline of AD, especially over an extended period of time. Although no control group was included, the present study otherwise demonstrates a widespread and enduring stoppage of progressive AD cognitive impairment through 2½ years by a new bio-engineering-based technology, transcranial electromagnetic treatment (TEMT). Moreover, we did not find any adverse effects in our limited group of AD patients over the extended period of this study. Although the present results are encouraging for TEMT’s ability to stop the progressive cognitive decline of AD, extended placebo-controlled clinical trials are necessary to establish that ability and to thus forward TEMT as perhaps the first AD therapeutic intervention capable of stopping or reversing this dreaded brain disease.

## 6. Patents

The following eight U.S. and International patent applications have resulted from the research performed in this manuscript, as submitted by NeuroEM Therapeutics, Inc., (Phoenix, Arizona, USA):

CIP No. 16/273,519, filed 2/12/19, and International PCT/US19/17650, filed 2/12/19, “Systems for Controlling Power to Differently Loaded Antenna Arrays”.

CIP 16/359,749, filed 3/20/19, and International PCT/US19/23244, filed 4/1/19, “Systems for Sensing Proper Emitter Array Placement”.

CIP No 16/936,152, filed 4/23/20, and International PCT/US20/029563, filed 7/22/20, “Transcranial Electromagnetic Treatment”.

CIP 16/865,250, filed 5/1/20, and International PCT/US2020/031192, filed 5/1/20, “Immunoregulation, Brain Detoxification, and Cognitive Protection by Electromagnetic Treatment”.

## Figures and Tables

**Table 1:** Subject demographics/characteristics.

Subject	1	2	3	4	5	Mean
Age	77	76	66	63	65	69.4
Gender	F	F	M	F	F	-----
ApoE Genotype	2/3	3/3	3/3	3/3	2/3	-----
MMSE Score	26	19	21	17	19	20.4
GDS Rating	4	4	3	5	4	4.0
Education	19	16	15	13	14	15.4
Anat. MRI Analysis	a,b	a,b	a	b	a	-----
PET AD Sign. ROI	1.10	1.06	1.10	1.32	1.40	1.20
Aβ1-42/t-tau Ratio	1.48	1.02	0.93	0.61	-----	1.01
**ADAS-cog13 Score**	**24.0**	**26.7**	**30.3**	**30.7**	**37.3**	**29.8**

Abbreviations: a, hippocampal/temporal lobe atrophy; b, global cortical atrophy; MRI, magnetic resonance imaging; PET, positron emission tomography; ROI, region of interest.

**Table 2:** Trajectory of change over time in cognitive scores (mixed-effects model) and baseline vs. 27–31 M comparison.

COGNITIVE DOMAIN	Est.	SEM	*p*-Value	Baseline vs. 27–31 M
ADAS-Cog Overall				***p*= 0.144** (t = −2.34)
Intercept	−0.7	0.29	0.091	
Change over time	0.02	0.02	**0.457**	
ADAS Immediate Recall				***p*= 0.423** (t = 1.00)
Intercept	0.11	1.21	0.931	
Change over time	−0.01	0.03	**0.848**	
Rey AVLT Sum of 5 Trials				***p*= 0.525** (t = 0.762)
Intercept	0.44	0.43	0.365	
Change over time	−0.03	0.03	**0.239**	
Rey AVLT Retroactive Interference				***p*= 1.000** (t = 0.000)
Intercept	0.66	0.48	0.246	
Change over time	−0.11	0.04	**0.051**	
MMSE				
Intercept	0.66	0.23	0.067	***p*= 0.287** (t = 1.44)
Change over time	−0.05	0.03	**0.266**	
ADL				***p*= 0.225** (t = 1.73)
Intercept	0.67	0.38	0.177	
Change over time	−0.06	0.03	**0.137**	
Digits Forward				***p*= 0.184** (t = 2.00)
Intercept	−0.13	0.38	0.754	
Change over time	0.05	0.03	**0.153**	
Digits Backward				***p*= 0.667** (t = 0.50)
Intercept	0.23	0.41	0.606	
Change over time	−0.03	0.03	**0.342**	
Cognitive composite				***p*= 0.328** (t = 1.28)
Intercept	0.17	0.2	0.456	
Change over time	−0.01	0.01	**0.399**	
Global Deterioration Scale (GDS)				***p*= 0.423** (t = −1.00)
(Qualitative, by caregiver)				

## Data Availability

The datasets used and/or analyzed during the current study are available from the corresponding author upon reasonable request.
